# Water and Constipation

**Published:** 1885-02

**Authors:** 


Water and Constipation.—Dr. Squibb, in his last *Ephemeris*, has a suggestive article on constipation. He thinks that most constipations are due to insufficient water in the fæcal mass. The individual does not ingest enough water to supply the blood with its seventy-nine per cent., therefore the blood absorbs it from the intestinal contents. "Drink water" is Dr. Squibb's motto. It is a good one, but water, as every one knows, will not cure habitual constipation in a very large percentage of cases.

